# Drought alters cowpea root endophytic fungal communities and functional characterization reveals plant growth-promoting potential of cultured endophytes

**DOI:** 10.1371/journal.pone.0360553

**Published:** 2026-11-11

**Authors:** Boshra Ahmed Halo, Mahmoud W. Yaish

**Affiliations:** Department of Biology, College of Sciences, Sultan Qaboos University, Muscat, Oman; University of Salento: Universita del Salento, ITALY

## Abstract

Drought is a major constraint to cowpea productivity, but its effects on root-associated endophytic fungi remain poorly understood. This study investigated how drought influences cowpea root endophytic fungal communities and evaluated the plant growth-promoting potential of cultured fungal endophytes. Internal transcribed spacer (ITS) amplicon sequencing revealed that drought significantly altered fungal community composition. Several fungal taxa, including *Aspergillus* and *Chaetomium*, showed numerical differences in abundance under drought, although these taxon-specific differences were not significant after correction for multiple comparisons. In parallel, 13 fungal endophytes were isolated from drought-treated cowpea roots and evaluated for plant growth-promoting traits. The isolates differed in 1-aminocyclopropane-1-carboxylate (ACC) deaminase activity and presumptive indole-3-acetic acid (IAA) production, and several promoted wheat seedling growth under polyethylene glycol (PEG)-induced osmotic stress. Selected isolates also enhanced okra pollen germination under osmotic stress. Together, these findings demonstrate that drought alters the cowpea root endophytic fungal community and identify cultured endophytes with promising plant growth-promoting activities. Further evaluation through direct inoculation of cowpea under drought is needed to determine their potential for improving crop performance under water-limited conditions.

## Introduction

Cowpea (*Vigna unguiculata*) is a staple legume of importance in semi-arid and drought-prone regions. It plays a central role in food security as an affordable and accessible source of dietary protein in many low- and middle-income countries [1]. In addition, cowpea contributes substantially to soil fertility through its symbiotic nitrogen fixation, making it particularly valuable in low-input agricultural systems [2]. Its importance is expected to grow as drought frequency and intensity increase under climate change scenarios. These trends highlight the need for sustainable strategies to maintain crop productivity under water-limited conditions, where drought-tolerant crops such as cowpea can play a critical role [3].

In plants, drought-induced changes in root architecture, osmotic balance, hormonal signaling, and oxidative stress can alter root exudation patterns and belowground carbon allocation, thereby influencing plant–microbe interactions in the rhizosphere and root-associated compartments [4].

Several studies on the mycobiome of major legumes have provided clear evidence that both edaphic factors and host identity shape fungal community assembly. For example, metagenomic and amplicon-based analyses in soybean demonstrate that soil properties and plant genotype influence the composition of root and rhizosphere fungal communities [5]. In cowpea, an ITS2-based survey characterized endophytic fungal communities across multiple cowpea tissues, establishing a foundational description of taxonomic composition and tissue specificity [6]. Additional cowpea studies have concentrated primarily on arbuscular mycorrhizal fungi (AMF), evaluating colonization dynamics and functional consequences for plant nutrition and growth [7].

While high-throughput ITS sequencing has been used to profile the cowpea mycobiome [6], to our knowledge, no prior ITS-based study has specifically assessed drought-driven shifts in the cowpea root endophytic fungal community. Comparable microbiome shifts under drought or other abiotic stress have, however, been reported in other legumes and crops [5,8–10]. Although recent research has demonstrated that drought alters rhizosphere and rhizoplane bacterial communities, with associated physiological effects on plants [8], comparable high-throughput ITS-based studies on the fungal community remain largely absent, highlighting a distinct gap in current understanding.

Abiotic stress is recognized as a deterministic driver of microbial community assembly, and drought has been shown to significantly alter the diversity and composition of plant-associated bacterial communities [11]. Fungal communities also respond to water limitation; shifts in root and rhizosphere fungal diversity under drought have been reported in non-leguminous systems [9,10]. Moreover, drought conditions may promote the enrichment of stress-tolerant and potentially beneficial taxa that contribute to host adaptation under water deficit, leading to broader plant microbiome syntheses [12]. Therefore, we hypothesize that drought functions as an environmental filter in cowpea, restructuring the endophytic root-associated fungal community and promoting taxa adapted to dry conditions.

Linking shifts in microbial community composition to functional traits is critical for interpreting the ecological significance of drought-induced microbiome changes and for identifying taxa with potential agronomic value [13]. Certain drought-enriched fungi and other root-associated microbes have been shown to exhibit plant growth–promoting properties, such as phytohormone production, modulation of stress ethylene via ACC deaminase activity, and enhancement of osmoprotectant pathways, thereby mitigating the negative effects of water deficit and improving host resilience [14].

Plant growth-promoting fungi are increasingly recognized as key components of the microbiome that enhance stress tolerance and support host development under adverse environmental conditions [12]. In this context, integrating high-throughput sequencing with culture-dependent isolation and phenotypic characterization enables a direct link between community composition and functional trait expression, facilitating the identification of beneficial strains with potential applications in drought-affected agroecosystems [15].

Building on evidence that drought alters root-associated microbial communities, isolating fungi from water-limited systems enables direct functional evaluation of taxa that persist under stress. Therefore, candidate isolates were screened in vitro for plant growth-promoting traits, including Salkowski-reactive indolic compounds (presumptive IAA) and ACC deaminase activity, followed by plant-based assays to evaluate their effects on host growth under control and PEG-induced osmotic stress conditions. Integrating ecological context with functional characterization strengthens the identification of strains with translational potential and represents an early, screening-level step toward the eventual development of fungal bioinoculants for drought-affected agroecosystems, pending field- and host-specific validation [16,17].

This study aimed to identify fungal taxa showing exploratory abundance trends between well-watered and drought conditions. In addition, we isolated candidate fungi exhibiting strong plant growth-promoting activity and evaluated their functional potential using wheat as a model host plant. To further assess bioactivity, fungal culture filtrates were tested for their effects on pollen grain germination in okra, using this as a complementary assay system.

## Materials and methods

### Experimental design and plant growth conditions

Two irrigation regimes were applied to investigate drought-driven changes in the diversity and composition of cowpea root endophytic fungal communities: a well-watered control (WP) and a drought-stressed treatment (DP). Each treatment included four biological replicates, each replicate consisting of pooled root samples of four plants grown together in a single 3-L pot. The samples analyzed in this study are the same biological samples previously used for bacterial microbiome analysis [18]. Here, we focus specifically on the fungal community using ITS sequencing.

Prior to sowing, cowpea seeds were surface-sterilized following a sequential sterilization protocol described by Oyebanji, Nweke [19], to minimize seed-borne microbial contamination. Sterilized seeds were planted in homogenized soil collected from the Al Seeb region, Muscat, Oman (23.6472°N, 58.1456°E). All pots and tools were disinfected before being used to reduce environmental contamination. Plants were cultivated under controlled conditions with a 16 h light/8 h dark photoperiod at 24 °C and irrigated with autoclaved distilled water to maintain soil moisture at field capacity during establishment. After six weeks, pots were randomly assigned to either the control or drought treatment. The drought-stressed pots received no irrigation for three weeks to impose a water deficit as previously described [20]. Soil moisture and electrical conductivity were monitored throughout the experiment using 5TE sensors and ECH2O software (METER Group, Pullman, WA, USA) to confirm treatment conditions. Soil moisture/EC monitoring and the drought-imposition protocol used here are shared with our companion studies on the bacterial component of this experimental system [18,20], which readers may consult for additional soil and plant physiological characterization where available; the present manuscript focuses specifically on the root endophytic fungal community and its culturable functional isolates.

### Root sampling and DNA extraction

Root tissues from each pot were carefully collected and pooled to form a single biological replicate, with each replicate representing material from four individual plants. Any soil particles attached to the roots were gently removed with a sterilized brush, and the roots were thoroughly rinsed under running tap water to remove any remaining debris. Root surface sterilization was performed using a stepwise chemical treatment adapted from Yaish, Antony [21]. Roots were sequentially immersed in 30% hydrogen peroxide (3 min), 70% ethanol (3 min), and 0.5% sodium hypochlorite (3 min). Sterilized roots were rinsed once with sterile water containing 10% Tween-20, followed by three additional rinses with sterile distilled water. Surface-sterilized root tissues were dried using sterile tissue paper, and total genomic DNA was extracted separately for each biological replicate using the DNeasy Plant Mini Kit (Qiagen, Hilden, Germany) following the manufacturer’s instructions. DNA quality and concentration were assessed using a NanoDrop spectrophotometer and confirmed by 1% agarose gel electrophoresis.

### Amplicon sequencing and bioinformatics analysis

The internal transcribed spacer 1 (ITS1) region was amplified from extracted DNA of endophytic fungal communities using the primers ITS1F (5′-CTTGGTCATTTAGAGGAAGTAA-3′) and ITS2 (5′-GCTGCGTTCTTCATCGATGC-3′). The resulting amplicons were subjected to high-throughput sequencing on an Illumina platform using 301-bp paired-end reads. Sequencing libraries were prepared separately for each biological replicate. Across the eight samples, raw sequencing generated 214,144–273,436 individual reads per sample, corresponding to 107,072–136,718 paired-end read pairs per sample.

Raw sequence data were processed using a standardized bioinformatics workflow. Forward and reverse reads were trimmed to 250 bp, and adapter and primer sequences were removed using Cutadapt (v3.2) [22]. Quality filtering, error correction, denoising, paired-end merging, and chimera removal were performed using DADA2 (v1.18.0) [23] to infer amplicon sequence variants (ASVs). Reads with expected errors ≥2 were excluded. Following error correction and denoising, paired-end reads were merged based on sequence overlap, and chimeric sequences were removed using the consensus method implemented in the “removeBimeraDenovo” function of DADA2.

Each ASV was taxonomically assigned by comparison against the National Center for Biotechnology Information (NCBI) Nucleotide (NT) database and the UNITE Community reference database. Sequence similarity searches were performed using BLAST+ (v2.9.0) and VSEARCH (v2.22.1), and the reference sequence with the highest similarity was used for taxonomic assignment. Only matches with query coverage >85% and sequence identity >85% were retained. Downstream ecological analyses were conducted using QIIME (v1.9.0) [24].

### Alpha and beta diversity analysis

Rarefaction analysis was performed using QIIME (v1.9.0) to evaluate whether sequencing depth was sufficient to adequately represent the observed fungal community diversity. Rarefaction curves were generated across increasing sequencing depths to assess the relationship between sequencing effort and alpha diversity. To account for differences in sequencing depth among samples, the dataset was normalized by subsampling to 79,285 sequences per sample, corresponding to the lowest number of non-chimeric sequences retained among the samples. The resulting normalized ASV dataset was used for downstream diversity analyses.

Alpha diversity metrics, including observed ASV richness, Shannon index, Gini-Simpson index, and phylogenetic diversity (PD whole tree), were calculated to assess within-sample richness, diversity, evenness, and phylogenetic structure. Multiple sequence alignment was performed using MAFFT (v7.475) [25], and a phylogenetic tree was constructed using FastTreeMP (v2.1.10). The Gini-Simpson index (1 − Σpᵢ²) increases with community diversity and evenness, whereas lower values indicate greater dominance by fewer taxa [26,27]. Mean values and standard deviations were calculated for each treatment group. Differences in alpha diversity between the control (WP) and drought (DP) treatments were assessed using independent-samples t-tests after evaluating the assumptions of normality and homogeneity of variance, with homogeneity of variance assessed using Levene’s test. Statistical significance was defined at *P* ≤ 0.05.

Beta diversity was assessed using Bray-Curtis, weighted UniFrac, and unweighted UniFrac distance metrics to evaluate differences in fungal community composition among samples. Relationships among samples were visualized using principal coordinate analysis (PCoA) and unweighted pair group method with arithmetic mean (UPGMA) clustering, and treatment effects were tested using permutational multivariate analysis of variance (PERMANOVA) with 9,999 permutations in PAST v5.0 [28]. Homogeneity of multivariate dispersion among groups was assessed using a permutation-based test of equal multivariate dispersions (PERMDISP) [29] using PAST v5.0, based on Bray-Curtis dissimilarities and 9,999 permutations. The same data transformation and Bray-Curtis dissimilarity measure used for the PERMANOVA were applied. Hierarchical clustering was performed using the unweighted pair-group method with arithmetic mean (UPGMA) based on the Bray–Curtis distance matrix, generating a clustering tree.

### Taxonomic composition analysis

Relative abundance percentages of endophytic fungal taxa were used to assess differences in community composition between well-watered (WP) and drought-stressed (DP) cowpea plants. Taxonomic data were organized and summarized at the family, genus, and species levels to evaluate treatment-associated shifts in fungal communities. The most abundant taxa were identified based on their relative contribution within each treatment. Differences in abundance of each ASV between the two groups (control vs. drought treatment) were identified using the Mann–Whitney U test (two-tailed) with a significance level of α = 0.05. Because 47 simultaneous per-ASV comparisons were performed, Benjamini–Hochberg false discovery rate (FDR) correction [30] was additionally applied; both raw and FDR-adjusted P-values (q-values) are reported in Table 2.

### FUNGuild-based ecological guild prediction of fungal ASVs

Functional annotation of fungal ASVs was performed using the FUNGuild online database [31]. The ASV table containing taxonomic assignments was processed in Python (version 3.14.2) using the pandas library. To ensure reliability of the functional classification, only assignments with confidence rankings of “Probable” and “Highly Probable” were retained, while entries categorized as “Possible” were excluded.

Fungal ASVs were classified according to their trophic modes, including Saprotroph, Pathotroph, and Symbiotroph, based on the FUNGuild annotations. The frequency of each trophic mode was calculated by counting occurrences within the trophic mode column, and ASVs assigned to multiple trophic strategies were included in all relevant categories.

### Isolation of culturable endophytic fungi

Endophytic fungi were isolated from surface-sterilized cowpea root tissues collected from drought-treated plants, following the method described by Halo, Al-Yahyai [32]. Briefly, root, shoot, and leaf tissues were thoroughly washed under running tap water and cut into small segments (approximately 5 mm long). The tissue segments were surface-sterilized by sequential immersion in 70% ethanol for 1 min, followed by 1% sodium hypochlorite for 1.5–2 min, and subsequently rinsed twice with sterile distilled water. Four sterilized tissue segments from each sample were aseptically placed onto Petri dishes containing 2.5% potato dextrose agar (PDA) supplemented with ampicillin (50 mg/L) and kanamycin (50 mg/L) to inhibit bacterial growth. The Petri dishes were incubated in the dark at 27 °C for 7 days and examined every two days for the emergence of endophytic fungal colonies. Emerging colonies were transferred to fresh PDA plates and repeatedly subcultured to obtain pure isolates. Isolation targeted the culturable fraction of the endophytic community present in drought-exposed root tissue and was not intended to specifically recover taxa showing abundance differences in the amplicon sequencing dataset (Table 2); the culture-dependent and culture-independent datasets are therefore complementary rather than confirmatory of one another, consistent with well-documented biases of culture-based recovery toward fast-growing, sporulating taxa [33,34].

### Molecular identification and phylogenetic analysis of fungal isolates

Genomic DNA of the endophytic fungal isolates was extracted from actively growing mycelia using a standard cetyltrimethylammonium bromide (CTAB)-based protocol as previously described [35,36], with minor modifications. Briefly, approximately 100 mg of fresh fungal mycelium was harvested, frozen in liquid nitrogen, and ground into a fine powder. The powder was incubated in CTAB extraction buffer (2% CTAB, 100 mM Tris-HCl, pH 8.0, 20 mM EDTA, 1.4 M NaCl, 0.2% β-mercaptoethanol) at 65°C for 30 min. DNA was then purified by chloroform: isoamyl alcohol extraction, precipitated with isopropanol, washed with 70% ethanol, air-dried, and resuspended in TE buffer. DNA quality and concentration were assessed using a NanoDrop spectrophotometer and verified by agarose gel electrophoresis.

To identify the fungal isolates, the internal transcribed spacer (ITS) region of rDNA was amplified by PCR using universal fungal primers ITS1 (5’-TCCGTAGGTGAACCTGCGG-3’) and ITS4 (5’-TCCTCCGCTTATTGATATGC-3’) [36,37]. PCR reactions were performed in a 25 µL volume containing 50 ng genomic DNA, 0.2 µM of each primer, and 2 × PCR master mix. Thermal cycling conditions were: initial denaturation at 95°C for 5 min; 35 cycles of 95°C for 30 s, 55°C for 30 s, and 72°C for 1 min; and a final extension at 72°C for 7 min. PCR products were verified by 1% agarose gel electrophoresis and purified using a commercial PCR purification kit.

Purified ITS amplicons were sequenced by the Sanger method using the sequencing facilities of Macrogen, South Korea, as a service provider. Sequence identities were determined using BLAST searches against the NCBI GenBank database, and the closest taxonomic affiliations of the isolates were determined based on ITS sequence similarity and phylogenetic placement.

The phylogenetic analysis of the ITS DNA sequences of the fungal isolates was constructed using the Neighbor-Joining method [38]. Evolutionary distances were calculated using the Kimura 2-parameter model, and expressed as the number of base substitutions per site. Rate variation among sites was modeled using a gamma distribution (shape parameter = 1). The ambiguous positions were removed using the pairwise deletion option, and 1,000 bootstrap replicates were used to assess branch support. All evolutionary analyses were performed using MEGA11 [39].

### Screening of endophytic fungal isolates for ACC deaminase activity

Endophytic fungal isolates were screened for ACC deaminase activity based on their ability to utilize ACC as a nitrogen source, following the method adapted for fungi by Wang, Tian [40]. The assay was used as a qualitative indicator of ACC deaminase activity. Fungal isolates were inoculated onto Foster (DF) minimal medium supplemented with 3.0 mM ACC as the sole nitrogen source and incubated under controlled conditions. Enzyme activity was assessed through the detection of α-ketobutyrate formation following ACC cleavage. Briefly, fungal cell extracts were reacted with 2,4-dinitrophenylhydrazine reagent under acidic conditions and incubated at 30 °C for 30 min to allow color development. The reaction was terminated by adding sodium hydroxide, which formed a colored complex. The intensity of the developed color was measured at 540 nm using a microplate reader spectrophotometer (Synergy MTX multi-mode reader, BioTek, Vermont, USA). ACC deaminase activity was evaluated qualitatively based on the observed color intensity relative to control reactions lacking ACC. Isolates were categorized using a relative scoring system (−, + , ++), where increased color intensity indicated higher ACC deaminase activity.

### Qualitative assessment of presumptive IAA production

Presumptive IAA production by endophytic fungal isolates was evaluated using a colorimetric assay based on Salkowski’s reagent [41]. Fungal isolates were cultured in potato dextrose broth (PDB) supplemented with L-tryptophan to induce IAA biosynthesis, while parallel PDB cultures without L-tryptophan served as controls. Fungal cultures were incubated in a shaking incubator at 30 °C and 120 rpm for seven days. After incubation, cultures were filtered and centrifuged to obtain cell-free culture supernatants. For presumptive IAA detection, 2 mL of culture filtrate was mixed with 1 mL of Salkowski’s reagent and incubated in the dark for 30 min. The development of pink-to-reddish coloration indicated the presence of indole-related compounds. Color development was measured at 530 nm using a microplate reader spectrophotometer (Synergy MTX multi-mode reader, BioTek, Vermont, USA). Presumptive IAA production was classified as − (≤0.15), + (>0.15 to 0.20), or ++ (>0.20), based on absorbance measurements relative to the negative control. ACC deaminase activity was classified as − (≤0.2177), + (>0.2177 to 0.30), or ++ (>0.30), based on absorbance measurements relative to the negative control. The semi-quantitative colorimetric assays [42,43] are effective for the comparative screening of the isolates’ functional potential. While focusing on relative activity levels, these categories provide a basis for identifying high-activity candidates that warrant further quantitative characterization, such as α-ketobutyrate production per mg protein or HPLC analysis.

### Evaluation of wheat seedling growth responses to endophytic fungal isolates under non-stress and PEG-induced osmotic stress conditions

The effects of endophytic fungal isolates on the early growth of wheat (*Triticum aestivum* L.) seedlings were evaluated using Petri dish bioassays under both non-stress and osmotic-stress conditions. Wheat seeds were surface-sterilized following the protocol described by [19] using a sequential ethanol-sodium hypochlorite treatment, followed by multiple rinses with sterile distilled water to eliminate external microorganisms. Wheat was selected as a standardized heterologous bioassay host for the initial functional screening of isolate bioactivity because its rapid germination and clear, quantifiable morphological read-outs (seedling shoot/root length) allow efficient, controlled comparison among the cowpea-derived isolates; this assay is not intended to predict the magnitude of isolate effects specifically in cowpea, which would require direct evaluation in that host.

Seed treatment with endophytic fungi was conducted following the protocol of Radhakrishnan, Khan [44] with minor modifications. Briefly, surface-sterilized seeds were immersed in 20 mL of a 2-week-old fungal culture grown in PDB for each isolate, then incubated on a rotary shaker at 120 rpm for 6 h. Control seeds were soaked in sterile PDB without fungal inoculation under identical conditions.

Following treatment, seeds were transferred to sterile, disposable Petri dishes lined with two layers of sterile filter paper. To assess osmotic stress responses, seedlings were irrigated with either sterile distilled water (non-stress condition) or a sterile polyethylene glycol solution (PEG 6,000; 15% w/v, 0.2 µm filtered). Petri dishes were incubated in the dark at 28 °C for 4 days. Each treatment consisted of two replicates, with 15 seeds per Petri dish. Control seeds were soaked in sterile PDB supplemented with PEG, without fungal inoculation, under the same conditions. At the end of the incubation period, shoot and root lengths were recorded for each seedling.

Statistical analyses of shoot and root length data were conducted for non-stress and PEG-induced osmotic stress conditions. Differences among treatments were assessed using one-way analysis of variance (ANOVA), followed by Fisher’s least significant difference (LSD) test for mean separation at *P* ≤ 0.05. Results were expressed as mean values ± standard deviation (SD).

### Assessment of okra pollen germination in response to endophytic fungal isolates under osmotic stress

The influence of endophytic fungal isolates on pollen germination was evaluated using okra (*Abelmoschus esculentus*) pollen under control and osmotic stress conditions. Brewbaker and Kwack (BK) germination medium [45] was prepared, and osmotic stress was induced by supplementing the medium with polyethylene glycol (PEG 6,000) at a concentration of 40%, a level previously reported to markedly inhibit pollen germination [46]. As with the wheat seedling assay above, okra was selected as a standardized heterologous bioassay host for functional screening of isolate bioactivity; this assay is likewise not intended to predict the magnitude of isolate effects specifically in cowpea.

Endophytic fungal culture filtrates were obtained from 10-day-old PDB cultures and sterilized by filtration through a 0.2 µm membrane. For each treatment, 0.5 µL of sterile culture filtrate was added to 0.5 mL of BK germination medium (0.1% v/v) prior to application. Control treatments consisted of BK medium, either without PEG (non-stress control) or with PEG, supplemented with an equal volume of sterile PDB in place of the fungal culture filtrate. Aliquots of the respective germination media were placed onto clean microscope slides, and freshly collected pollen grains were evenly dispersed directly onto the surface of the medium on each slide. The slides were positioned inside moistened Petri dishes to maintain high humidity and prevent desiccation. Incubation was carried out in the dark at 28 °C for 24 h, following [47]. After incubation, pollen germination was evaluated microscopically by scanning the entire slide for each treatment. A pollen grain was considered germinated when the pollen tube length exceeded the diameter of the pollen grain. Statistical differences between fungal treatments and their corresponding controls under each condition were analyzed using pairwise two-proportion Z-tests. Treatments sharing the same subscript letter were considered not significantly different at P ≤ 0.05. Statistical analyses were performed using Statistical Package for the Social Sciences (SPSS) [48].

### Statistical analysis

Unless otherwise noted below, statistical analyses were performed using SPSS v21.0 [48]. Alpha diversity indices (Shannon index, Gini–Simpson index, PD whole-tree, and ASV richness) were compared between well-watered (WP) and drought-stressed (DP) treatments using an independent-samples t-test. Homogeneity of variances was assessed using Levene’s test prior to analysis. Results are presented as mean ± standard deviation (SD), and statistical significance was set at *P* ≤ 0.05.

Beta diversity was assessed using Bray-Curtis dissimilarity matrices. Principal Coordinate Analysis (PCoA) was used to visualize group separation, and one-way PERMANOVA with 9,999 permutations was used to test the significance of treatment effects. UPGMA hierarchical clustering was performed to assess patterns of sample similarity. Homogeneity of multivariate dispersion was assessed using PERMDISP with 9,999 permutations. Beta-diversity ordination, PERMANOVA, and PERMDISP were performed in PAST v5.0 [28].

For Petri dish growth assays, shoot and root lengths were analyzed using one-way ANOVA followed by Fisher’s least significant difference (LSD) test to separate means at *P* ≤ 0.05. For pollen germination experiments, pairwise two-proportion Z-tests were applied to compare each fungal treatment with its corresponding control. Treatments sharing the same subscript letter were considered not significantly different at *P* ≤ 0.05.

## Results

### High-quality sequencing data obtained from cowpea endophytic fungal communities

A total of 995,291 paired-end read pairs were generated across the eight cowpea root samples, ranging from 107,072–136,718 read pairs per sample. Following adapter and primer trimming, quality filtering, denoising, paired-end merging, and chimera removal, each sample retained between 79,285 and 107,694 high-quality non-chimeric sequences suitable for downstream analysis. The relatively uniform sequencing depth across well-watered (WP) and drought-stressed (DP) samples provided sufficient sequence coverage for subsequent diversity and community composition analyses.

### Alpha diversity indices of endophytic fungal communities associated with the roots under control (WP) and drought (DP) conditions

Rarefaction curves showed progressive stabilization of diversity estimates with increasing sequencing depth, with the Shannon diversity curves reaching a clear plateau across all samples. Samples were normalized to 79,285 sequences per sample, corresponding to the lowest number of non-chimeric sequences retained among the samples. The stabilization of the rarefaction curves before the maximum sequencing depth indicated that sequencing effort was sufficient to adequately represent the observed fungal community diversity (S1 Fig). Alpha diversity analysis revealed no significant differences between control (WP) and drought (DP) treatments for the Shannon index, phylogenetic diversity (PD whole tree), or ASV richness (*P* > 0.05; Table 1). In contrast, the Gini-Simpson index was significantly lower under drought than under control conditions (*P* ≤ 0.05).

**Table 1:** Comparison of alpha diversity metrics (ASVs, Shannon, Gini–Simpson, and PD whole-tree) between control and drought treatments (data are represented as Mean ± SD). Different letters next to the numerical values in the table indicate statistically significant differences among the corresponding entries.

Treatments	Shannon	Gini-Simpson	PD_whole_tree	ASVs
WP	1.63 ± 0.11a	0.48 ± 0.03a	8.23 ± 0.73a	56.25 ± 3.86a
DP	1.58 ± 0.21a	0.38 ± 0.05b	8.3 ± 1.21a	58.25 ± 5.5a

### Beta diversity reveals significant shifts in endophytic fungal communities under drought

WP and DP samples were clearly separated in the PCoA plot, with the four replicates within each treatment forming tight clusters. The first two axes explained 76.8% of the total variation, capturing 54.3% and 22.5% of the total variation on Coordinates 1 and 2, respectively (Fig 1A). The observed differences between the WP and DP groups were statistically significant, as confirmed by a one-way PERMANOVA (F = 6.455, *P* = 0.0286, 9999 permutations), demonstrating that drought stress significantly alters fungal community structure. Multivariate dispersion did not differ significantly between groups (PERMDISP, Bray-Curtis: F_1,6_ = 1.708, *P* = 0.2614; 9,999 permutations), providing no evidence that the observed PERMANOVA differences were attributable to heterogeneity of within-group dispersion. Consistent with these results, UPGMA clustering showed that replicates within each treatment group were clustered closely together. At the same time, WP and DP formed distinct clusters, indicating treatment-dependent differences in community composition, with WP replicates clustering more tightly than DP, as evidenced by shorter branch lengths in the tree (Fig 1B).

**Fig 1:**
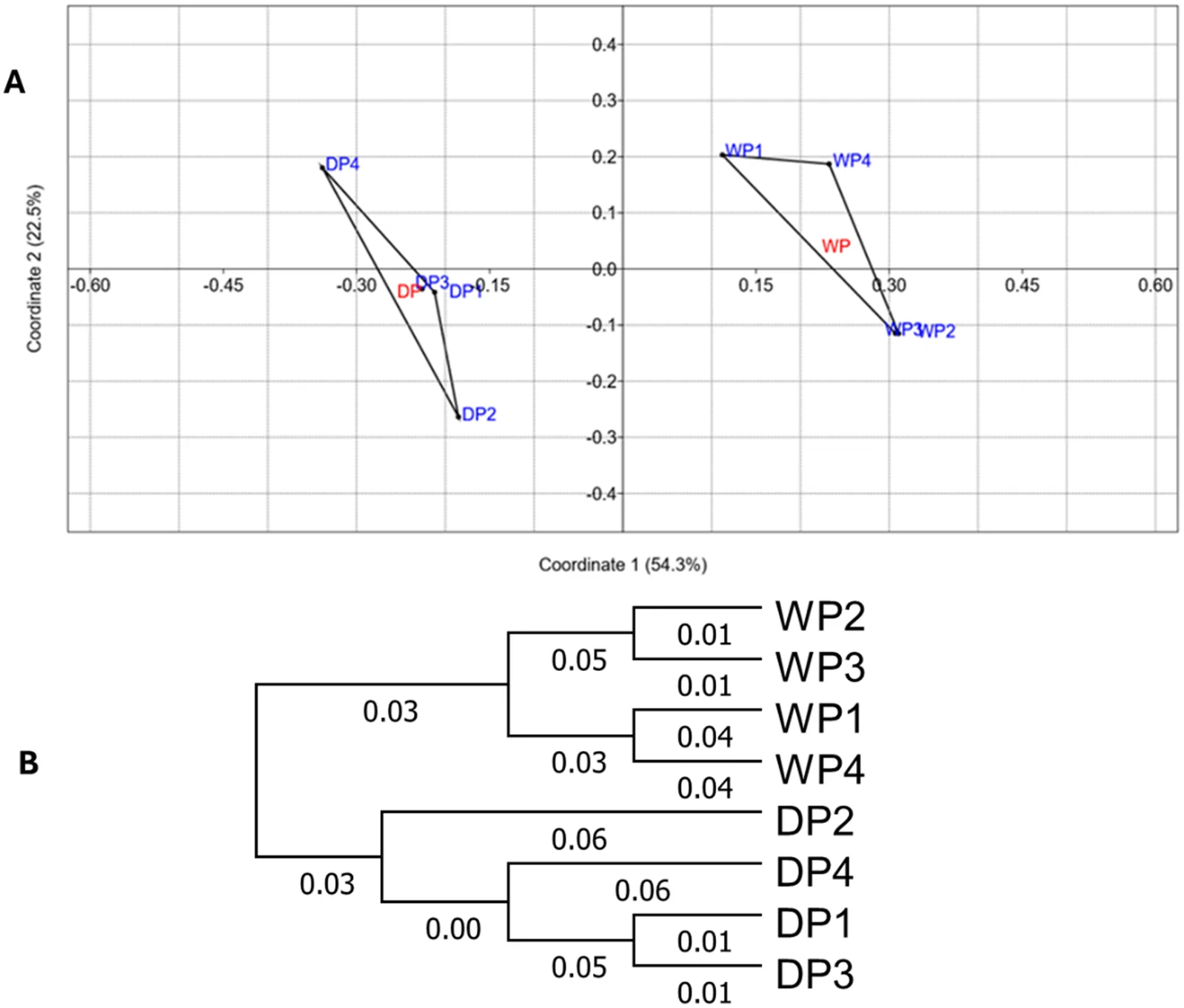
Beta diversity of endophytic fungal communities across eight independent samples from cowpea plants under control (WP1–WP4) and drought (DP1–DP4) conditions, based on taxa abundance. Principal coordinate analysis (PCoA) illustrating the separation of endophytic fungal communities associated with cowpea under well-watered (WP) and drought-stressed (DP) conditions (A). Group separation was significant based on one-way PERMANOVA (F = 6.455, *P* = 0.0286, 9999 permutations). UPGMA hierarchical clustering of samples based on the Bray–Curtis distance matrix. The tree shows the grouping of replicates within the same treatment group (B).

### Drought stress alters the taxonomic composition of the endophytic fungal community

The ASVs obtained from the WP and DP samples were taxonomically classified across multiple hierarchical levels (phylum, class, order, family, genus, and species) to enhance a comprehensive comparison of microbial community composition and structure between the two groups (S1 Table and Fig 2). Across taxonomic levels, differences in the observed community profiles were evident between WP and DP samples, consistent with the community-level separation detected by PERMANOVA.

**Fig 2:**
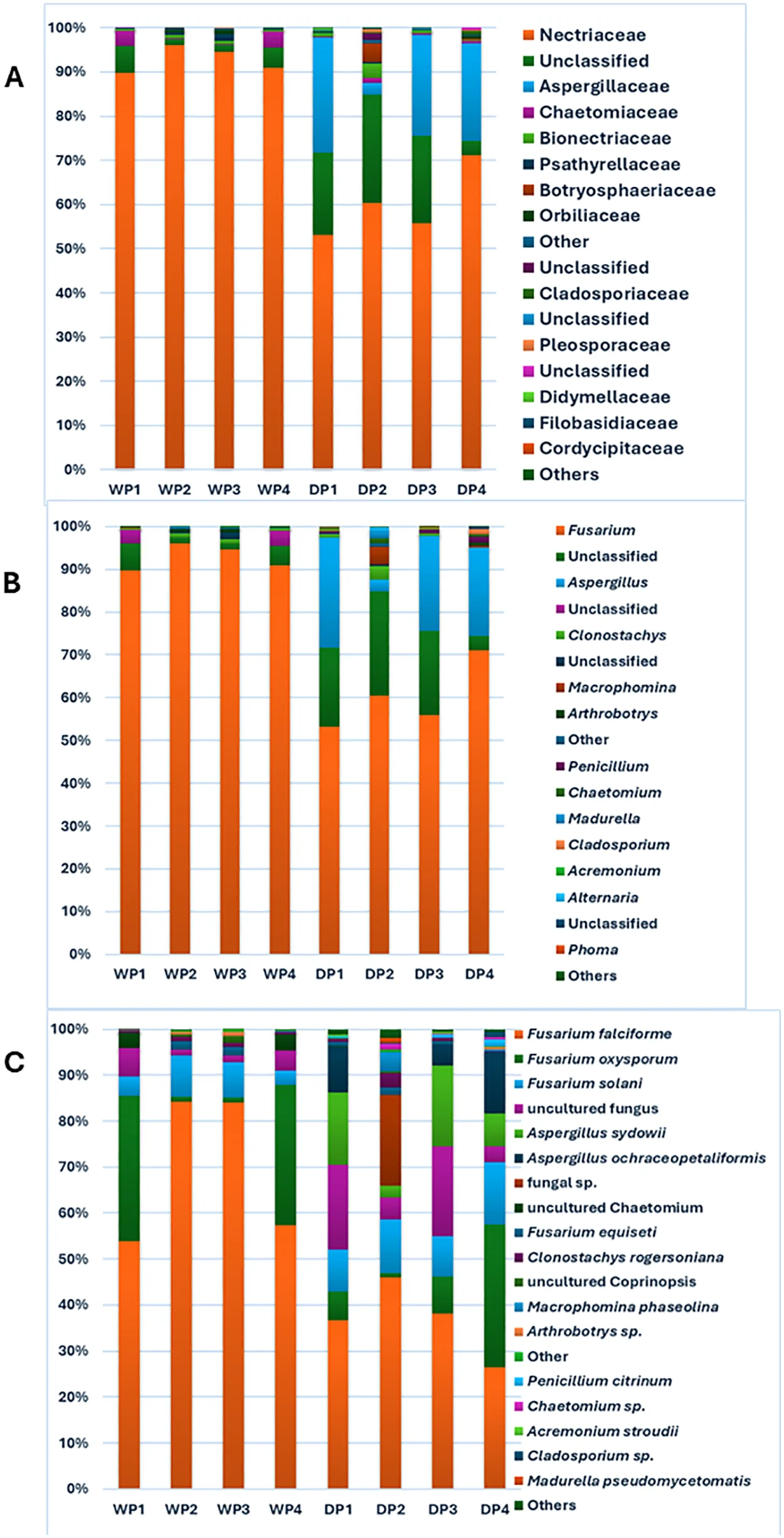
Relative taxonomic abundance of the dominant fungal taxa associated with cowpea under well-watered (WP) and drought-stressed (DP) conditions at the family (A), genus (B), and species (C) levels, where WP1–WP4 and DP1–DP4 represent four biological replicates per treatment.

At the family level (Fig 2A), endophytic fungal communities in well-watered plants were predominantly Nectriaceae, which accounted for most of the relative abundance in WP samples. In contrast, drought-stressed plants showed a more heterogeneous family-level composition, with reduced Nectriaceae dominance and increased contributions from other families, such as Aspergillaceae, along with higher proportions of unclassified and low-abundance families, suggesting a restructuring of fungal communities under drought conditions.

At the genus level (Fig 2B), *Fusarium* was the most abundant genus across both treatments; however, its relative abundance was notably higher in well-watered plants than in drought-stressed plants. Under drought conditions, a broader range of genera, including *Aspergillus, Macrophomina*, and *Chaetomium*, showed numerically higher relative abundance.

At the species level (Fig 2C), fungal community composition differed markedly between treatments. *Fusarium falciforme* was the dominant fungal species in both conditions, but exhibited substantially higher relative abundance in well-watered plants than in drought-stressed plants. *F. oxysporum* followed a similar trend, with reduced abundance under drought stress. In contrast, *Aspergillus sydowii* and *A. ochraceopetaliformis* showed numerically higher relative abundance under drought conditions and were absent or nearly absent in well-watered samples. Additionally, drought samples showed higher proportions of uncultured fungi and rare taxa, indicating increased species-level community heterogeneity.

The relative abundance of endophytic fungal taxa associated with cowpea plants showed limited numerical differences between control and drought conditions (Tables 2 and S1). A small subset of taxa showed nominally significant differences in abundance in response to drought stress based on uncorrected comparisons (*P* ≤ 0.05); however, none of these differences remained statistically significant after correction for multiple comparisons*. Aspergillus sydowii* and *Chaetomium* sp. showed nominally higher abundance under drought conditions (*P* = 0.018 and 0.020, respectively), whereas *Fusarium falciforme* showed nominally lower abundance under drought stress (*P* = 0.021). In addition, *Aspergillus ochraceopetaliformis* and *Penicillium citrinum* were detected only under drought conditions and showed nominal differences relative to the control (*P* = 0.047). After Benjamini-Hochberg correction for the 47 simultaneous ASV-level comparisons (Table 2), none of these taxa remained statistically significant (BH-FDR-adjusted q ≥ 0.33 for all comparisons). Accordingly, these taxon-level patterns should be regarded as exploratory numerical or nominal trends rather than evidence of statistically supported differential abundance. In contrast, the community-level PERMANOVA indicated a significant difference in fungal community composition between treatments, with no significant difference in multivariate dispersion between groups (PERMDISP: F_1,6_ = 1.708, *P* = 0.2614).

**Table 2:** ASV-level abundance comparisons of endophytic fungal taxa associated with cowpea under well-watered (WP) and drought-stressed (DP) conditions, ranked by raw *P*-value from two-tailed Mann–Whitney U tests. Mean relative abundances (± SD) were calculated for each ASV in the four ASV libraries per treatment. False Discovery Rate (FDR) q-values were adjusted using the Benjamini–Hochberg procedure.

ASV	Mean±SD (WP)	Mean±SD (DP)	*P*-Value	FDR q-value
*Aspergillus sydowii*	1 ± 2	1440 ± 1182.58	0.018	0.329
*Chaetomium* sp.	12.5 ± 1.91	54.5 ± 40.01	0.020	0.329
*Fusarium falciforme*	14581 ± 4026.12	4407.25 ± 1565.91	0.021	0.329
*Aspergillus ochraceopetaliformis*	0 ± 0	822.25 ± 653.72	0.047	0.440
*Penicillium citrinum*	0 ± 0	68.5 ± 52.88	0.047	0.440
*Aspergillus proliferans*	0 ± 0	2.5 ± 2.89	0.127	0.440
*Cladosporium harfordiae*	0 ± 0	15.5 ± 22.65	0.131	0.440
*Cladosporium* sp.	0 ± 0	26 ± 43.17	0.131	0.440
*Phoma* sp.	0 ± 0	11.25 ± 15.13	0.131	0.440
*Penicillium aeneum*	1.75 ± 2.36	0 ± 0	0.131	0.440
*Lecanicillium* sp.	0 ± 0	2.75 ± 3.4	0.131	0.440
*Sordariales* sp.	8.75 ± 10.11	0 ± 0	0.131	0.440
uncultured Sterigmatomyces	0 ± 0	3 ± 3.56	0.131	0.440
*Cystobasidium* sp.	0 ± 0	1.75 ± 2.36	0.131	0.440
uncultured Coprinopsis	113.75 ± 152.1	11.25 ± 22.5	0.166	0.457
*Lasiodiplodia* sp.	1.5 ± 3	0 ± 0	0.317	0.457
*Exserohilum rostratum*	0 ± 0	2.75 ± 5.5	0.317	0.457
*Penicillium cinnamopurpureum*	0 ± 0	1 ± 2	0.317	0.457
*Penicillium corylophilum*	0 ± 0	1 ± 2	0.317	0.457
*Penicillium daleae*	0.75 ± 1.5	0 ± 0	0.317	0.457
*Cephaliophora irregularis*	2.5 ± 5	0 ± 0	0.317	0.457
*Sordariomycetes* sp.	0 ± 0	11.75 ± 23.5	0.317	0.457
*Parengyodontium album*	0 ± 0	0.75 ± 1.5	0.317	0.457
*Fusarium delphinoides*	1.25 ± 2.5	0 ± 0	0.317	0.457
*Madurella pseudomycetomatis*	0 ± 0	25.5 ± 51	0.317	0.457
*Chaetomiaceae* sp.	0 ± 0	0.75 ± 1.5	0.317	0.457
*Botryotrichum piluliferum*	0.25 ± 0.5	0 ± 0	0.317	0.457
*Sterigmatomyces halophilus*	0.75 ± 1.5	0 ± 0	0.317	0.457
*Cystobasidium minutum*	0 ± 0	1.5 ± 3	0.317	0.457
*Naganishia albida*	0 ± 0	2.75 ± 5.5	0.317	0.457
*Naganishia globosa*	0 ± 0	1.75 ± 3.5	0.317	0.457
*Ascomycota* sp.	2.75 ± 3.77	0.5 ± 1	0.321	0.457
*Arthrobotrys* sp.	68.75 ± 84.51	14.5 ± 29	0.321	0.457
*Macrophomina phaseolina*	11 ± 12.94	104.25 ± 188.85	0.375	0.518
uncultured fungus	678.75 ± 443.22	1590.25 ± 1422.83	0.386	0.518
*Cutaneotrichosporon debeurmannianum*	1.75 ± 2.36	0.75 ± 1.5	0.508	0.663
*Acremonium stroudii*	7.25 ± 10.87	24.5 ± 28.34	0.538	0.663
uncultured *Chaetomium*	346 ± 408.79	1.75 ± 2.36	0.538	0.663
*Clonostachys rogersoniana*	134.5 ± 58.09	127.75 ± 118.91	0.564	0.663
*Fusarium oxysporum*	3259.25 ± 3521.22	1194 ± 1037.81	0.564	0.663
*Fusarium equiseti*	199.75 ± 230.73	102.25 ± 70.49	0.767	0.879
*Microascus rothbergiorum*	0.75 ± 1.5	1.75 ± 3.5	0.850	0.908
*Madurella tropicana*	3.75 ± 7.5	12.5 ± 25	0.850	0.908
*Rhodotorula mucilaginosa*	1.5 ± 3	1.75 ± 3.5	0.850	0.908
fungal sp.	3.25 ± 3.95	468.5 ± 934.34	1.000	1.000
*Alternaria tenuissima*	4.25 ± 5.32	20.5 ± 37.74	1.000	1.000
*Fusarium solani*	1233 ± 600.1	1221.75 ± 130.4	1.000	1.000

### FUNGuild-based ecological guild prediction of fungal ASVs

Metabolic functional prediction using sequence data allows inference of a community’s potential metabolic activities and biological functions when direct functional measurements are unavailable. Ecological guild prediction using sequence-based taxonomic data allows inference of potential trophic strategies when direct functional measurements are unavailable. Functional annotation using FUNGuild revealed that saprotrophs were the dominant trophic mode among the endophytic fungal community associated with cowpea, followed by pathotrophs and symbiotrophs (Fig 3). Saprotrophic fungi accounted for the largest proportion of the annotated ASVs, whereas pathotrophic and symbiotrophic fungi were detected at lower frequencies. These results indicate that saprotrophic functional groups predominate in the endophytic fungal community colonizing cowpea root tissues under drought stress.

**Fig 3:**
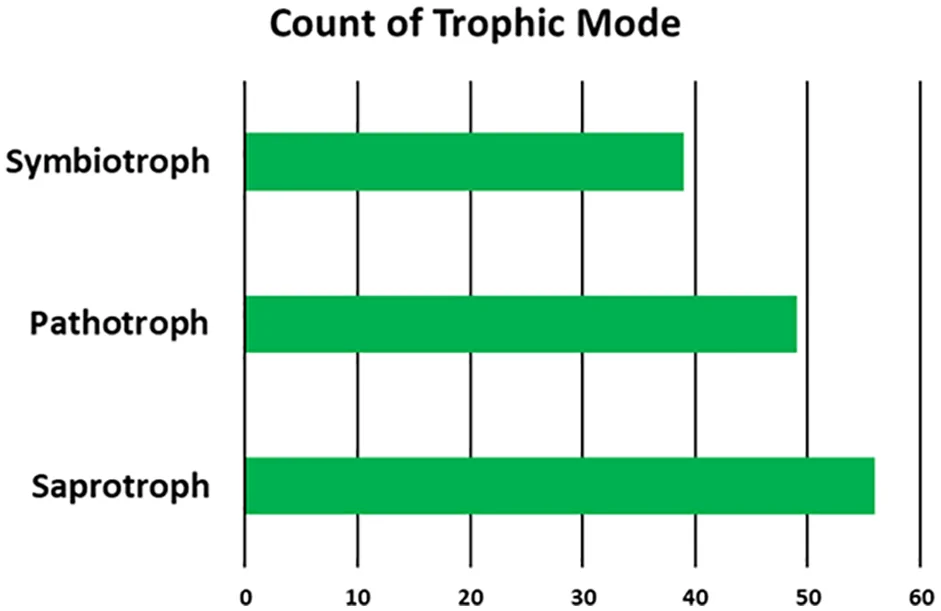
FUNGuild-based prediction of fungal trophic modes, showing the distribution of saprotrophic, pathotrophic, and symbiotrophic assignments among annotated fungal ASVs.

### Identification and phylogenetic analysis of the isolated endophytic fungi

Fungal isolation was conducted to obtain pure cultures for accurate taxonomic identification and to enable subsequent functional characterization of endophytic strains associated with cowpea under drought stress conditions. A total of 13 fungal strains were isolated and classified into three genera: *Fusarium*, *Aspergillus*, and *Penicillium*. At the species level, four distinct species were identified, namely *Fusarium falciforme*, *Aspergillus venezuelensis*, *Penicillium amphipolaria*, and *Penicillium singorense*. The isolates showed closest species-level affiliations with four taxa: *Fusarium falciforme*, *Aspergillus venezuelensis*, *Penicillium amphipolaria*, and *Penicillium singorense* (Table 3).

**Table 3:** Isolated fungal strains and their closest taxonomic affiliations based on ITS rDNA sequence analysis.

Strain	Closest Species	Identity (%)	GenBank Accession	Strain classification	GenBank deposit number
1F	*Fusarium falciforme*	100	NR_164424.1	*Fusarium falciforme*	PZ091415
2F	*Aspergillus venezuelensis*	97.88	NR_172042.1	*Aspergillus venezuelensis*	PZ091416
3F	*Penicillium amphipolaria*	100.00	NR_158827.1	*Penicillium amphipolaria*	PZ091417
4F	*Penicillium singorense*	99.79	NR_137877.1	*Penicillium singorense*	PZ091418
6F	*Fusarium falciforme*	100.00	NR_164424.1	*Fusarium falciforme*	PZ091419
7F	*Penicillium amphipolaria*	100.00	NR_158827.1	*Penicillium amphipolaria*	PZ091420
8F	*Penicillium singorense*	100.00	NR_137877.1	*Penicillium singorense*	PZ091421
9F	*Penicillium singorense*	99.56	NR_137877.1	*Penicillium singorense*	PZ091422
10F	*Fusarium falciforme*	99.77	NR_164424.1	*Fusarium falciforme*	PZ091423
11F	*Penicillium singorense*	99.78	NR_137877.1	*Penicillium singorense*	PZ091424
12F	*Penicillium singorense*	98.68	NR_137877.1	*Penicillium singorense*	PZ091425
13F	*Penicillium singorense*	100.00	NR_137877.1	*Penicillium singorense*	PZ091426
14F	*Fusarium falciforme*	100.00	NR_164424.1	*Fusarium falciforme*	PZ091427

Phylogenetic analysis revealed four distinct clades among the endophytic isolates. Six isolates (4F, 8F, 9F, 11F, 12F, and 13F) clustered with *Penicillium singorense*, forming the largest group. Four isolates (1F, 6F, 10F, and 14F) grouped with *Fusarium falciforme*, while two isolates (3F and 7F) clustered with *Penicillium amphipolaria*, and one isolate (2F) formed another clade corresponding to *Aspergillus venezuelensis*. The clustering pattern and high bootstrap values support separating the isolates into these four taxonomic groups (Fig 4).

**Fig 4:**
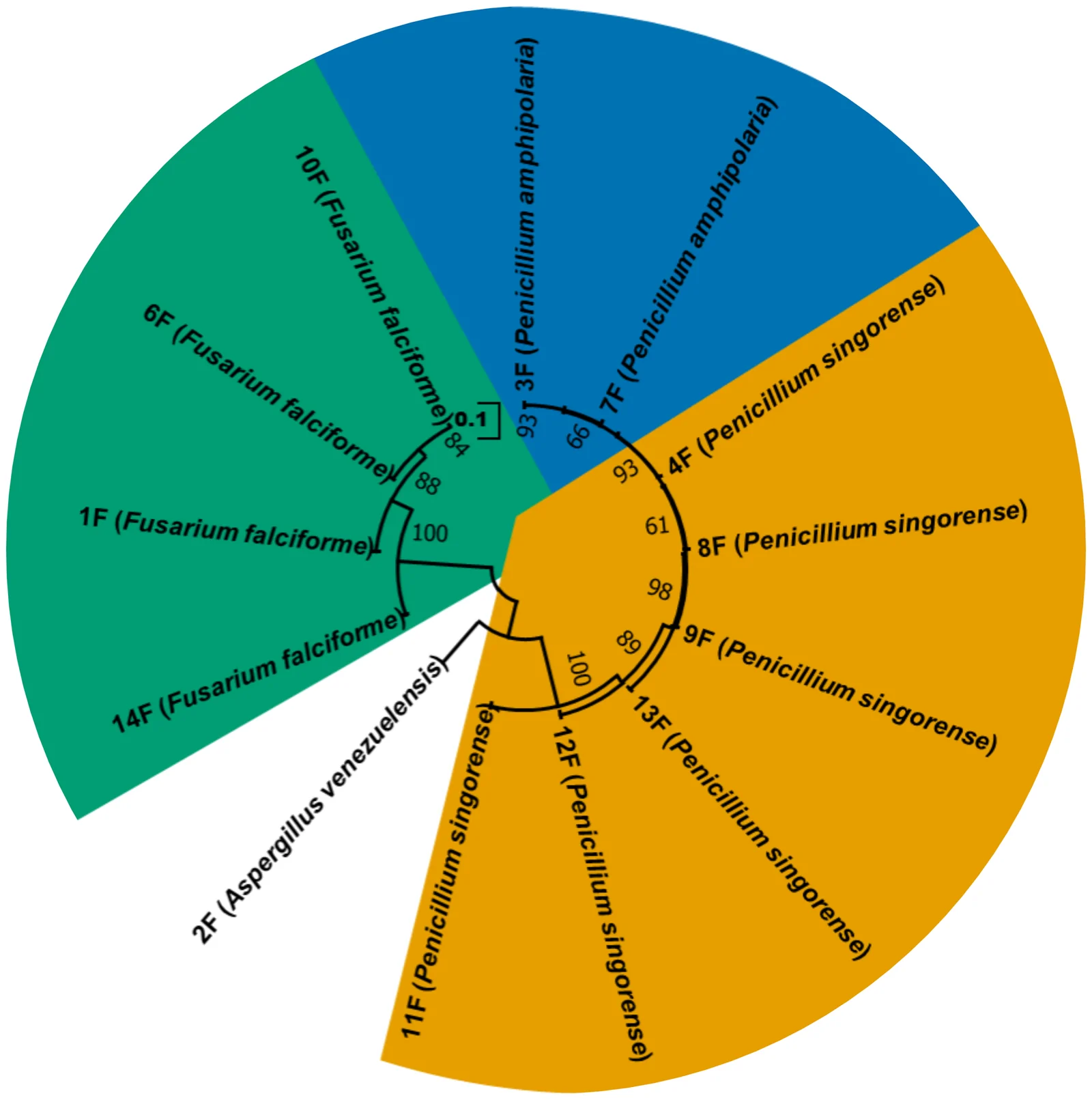
Phylogenetic tree constructed using the Neighbor-Joining method based on Kimura 2-parameter distances from ITS DNA sequences of the endophytic fungal isolates. Four distinct clades were identified among the isolates, each shaded in a different color in the tree. The clustering pattern and bootstrap support (1,000 replicates) support the separation of the isolates into four taxonomic groups corresponding to their closest species-level affiliations.

### Diversity of plant growth-promoting biochemical traits among endophytic fungal isolates

ACC deaminase and presumptive IAA activities were evaluated in the isolated strains to assess their potential to modulate plant stress responses and enhance host growth through key physiological and biochemical mechanisms (S2 Fig and S2 Table). Qualitative biochemical characterization revealed marked variation in ACC deaminase activity and presumptive IAA production among the endophytic fungal isolates (Table 4). ACC deaminase activity was detected in several isolates, with 2F showing strong activity (++), while isolates 1F, 6F, 9F, 11F, and 14F showed moderate activity (+). In contrast, presumptive IAA production was limited to a subset of isolates, with strong production (++) observed only in 1F, and moderate production (+) detected in 6F and 14F. The remaining isolates did not exhibit detectable presumptive IAA production. Notably, 1F, 6F, and 14F exhibited both ACC deaminase activity and presumptive IAA production.

**Table 4:** Qualitative assessment of ACC deaminase activity and presumptive IAA production in endophytic fungal isolates. Activity strength is indicated by the number of ‘+’ symbols; more ‘+’ signs denote higher activity.

Isolate	ACC Deaminase Activity	Presumptive IAA
**1F**	+	++
**2F**	++	−
**3F**	−	−
**4F**	−	−
**6F**	+	+
**7F**	−	−
**8F**	−	−
**9F**	+	−
**10F**	−	−
**11F**	+	−
**12F**	−	−
**13F**	−	−
**14F**	+	+
**Control**	−	−

### Endophytic fungal isolates promote wheat seedling growth under PEG-induced osmotic stress

We tested endophytic fungi isolated from cowpeas for their ability to promote wheat seedling growth under both non-stress and PEG-induced osmotic stress conditions. The isolates evaluated below were obtained from cowpea roots under drought stress (Table 3); wheat seedlings served solely as a standardized heterologous host for functional bioassay of isolate bioactivity and were not themselves exposed to drought or sampled for endophytes. Under non-stress conditions, most endophytic fungal isolates did not significantly affect shoot length, as most treatments fell into the same statistical groupings as the control (Fig 5A). However, isolate 10F significantly reduced shoot length compared to the control, indicating a clear inhibitory effect on shoot growth. Similarly, root length did not differ significantly between the control and any fungal treatment, with extensive overlap in statistical groupings across treatments (Fig 5B). Although numerical differences were observed, they were not statistically significant relative to the control.

**Fig 5:**
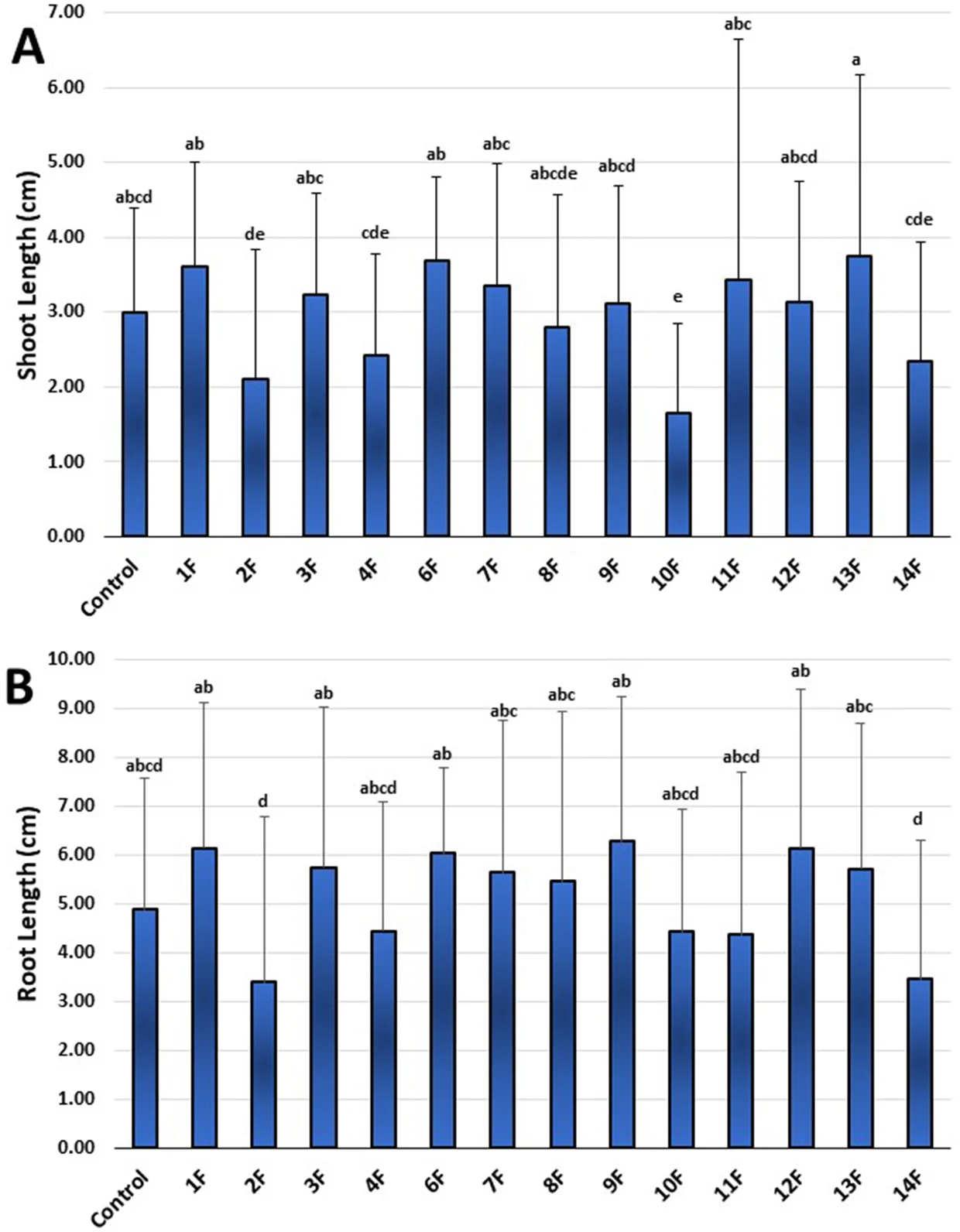
Growth responses of wheat seedlings to different endophytic fungal isolates under non-stress conditions in Petri dish assays, measured as shoot (A) and root (B) lengths. The control treatment consisted of seeds treated with sterile PDB. Bars represent mean values ± SD. Statistical differences among treatments were determined using one-way ANOVA followed by the LSD test at *P* ≤ 0.05; different letters above the bars indicate significant differences.

Under PEG-induced osmotic stress, endophytic fungal inoculation significantly affected wheat seedling shoot length (Fig 6A). Isolate 10F produced the greatest increase, significantly exceeding both the control and all other treatments (*P* ≤ 0.05). Isolates 3F, 11F, and 12F also resulted in significantly longer shoots than the control, while the remaining treatments did not differ significantly from the control. Root length of wheat seedlings under PEG-induced osmotic stress was significantly influenced by endophytic fungal inoculation (Fig 6B). Isolates 2F and 11F significantly increased root length compared to the control. Among these, 2F exhibited the strongest stimulatory effect on root elongation. However, the remaining treatments overlapped statistically with the control and did not significantly affect root length.

**Fig 6:**
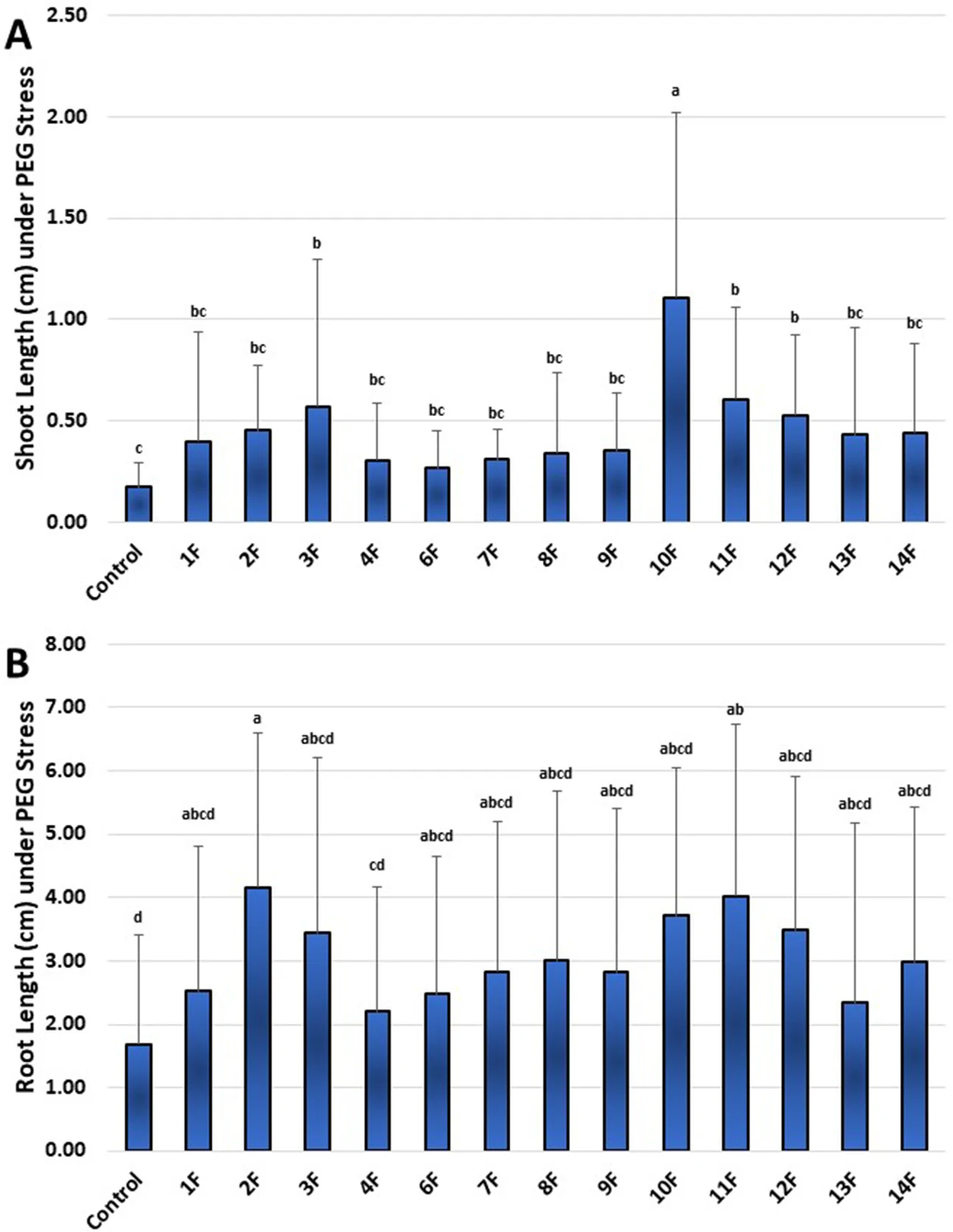
Growth responses of wheat seedlings to different endophytic fungal isolates under PEG-induced osmotic stress conditions in Petri dish assays, measured as shoot (A) and root (B) lengths. The control treatment consisted of seeds treated with sterile PDB supplemented with 15% PEG 6,000. Bars represent mean values ± SD. Statistical differences among treatments were determined using one-way ANOVA followed by the LSD test at *P* ≤ 0.05; different letters above the bars indicate significant differences.

### Endophytic fungi enhance okra pollen germination under osmotic stress

Endophytic fungi isolated from water-stressed cowpea roots were tested for their effects on okra pollen germination under osmotic stress to evaluate their potential to enhance reproductive resilience under water-limiting conditions. Under control conditions (Brewbaker & Kwack medium), inoculation with isolates 1F, 2F, and 6F resulted in significantly higher (*P* ≤ 0.05) pollen germination percentages (88.4–89.4%) compared with the control treatment (65.9%). In contrast, pollen germination was significantly reduced relative to the control in the presence of isolates 10F, 11F, and 12F.

Under PEG-induced osmotic stress, control pollen germination was markedly reduced (18%). However, isolates 1F, 2F, 3F, 6F, 7F, 8F, 11F, 13F and 14F significantly alleviated this inhibition, maintaining higher germination percentages (63.5%, 70.1%, 64%, 63.4%, 54.5%, 60.4%, 66.2%, 42.5%, and 73.3%, respectively), whereas germination in the presence of isolates 4F, 9F, 10F, and 12F was not significantly different from the control with point estimates numerically lower for 4F (11.8%) and 10F (4.5%) but numerically higher for 9F (34.3%) and 12F (22.6%) relative to the control (18.0%). Significant differences were determined using pairwise Two-proportion Z-tests (*P* ≤ 0.05), as indicated in Table 5.

**Table 5:** Germination (%) of okra pollen grains under control conditions (Brewbaker & Kwack medium) and under osmotic stress (Brewbaker & Kwack medium + PEG 6,000) for different treatments. Values represent the percentage of germinated pollen per treatment. Subscript letters indicate statistical groups within each column: treatments sharing the same letter are not significantly different at *P* < 0.05 based on pairwise Two-proportion Z-tests (Crosstabs in SPSS).

Treatment	Pollen germination (%) under control conditions	Pollen germination (%) under osmotic stress (PEG 6,000)
**1F**	89.4 a	63.5 a,b,c,d
**2F**	88.4 a,b	70.1 d
**3F**	84.7 a,b,c	64.0 a,b,c,d
**4F**	40.3 d	11.8 e
**6F**	88.4 a,b	63.4 a,b,c,d
**7F**	39.1 d	54.5 a,b,c,d
**8F**	35.9 d	60.4 a,b,c,d
**9F**	43.0 d	34.3 c,g,h
**10F**	15.0 e	4.5 e
**11F**	10.8 e	66.2 a,b,d
**12F**	6.7 e	22.6 e,f,g,h
**13F**	44.0 d	42.5 b,c,f,g
**14F**	71.4 b,c	73.3 a,d
**Control**	65.9 c	18.0 e,h

## Discussion

Cowpeas host diverse endophytic fungal communities that can enhance plant tolerance to environmental stress through complex physiological and biochemical interactions, which are affected by factors such as drought [6]. In the present study, alpha-diversity analysis indicated that drought did not substantially affect taxonomic richness or phylogenetic diversity, as most diversity indices did not differ significantly between treatments. However, the Gini-Simpson index was significantly lower under drought, indicating a change in community evenness. This result should be considered together with the beta-diversity analysis, which demonstrated a significant difference in overall fungal community composition between well-watered and drought-treated plants. Importantly, multivariate dispersion did not differ significantly between treatments (PERMDISP: F_1,6_ = 1.708, P = 0.2614), providing no evidence that the significant PERMANOVA result was attributable to unequal within-group dispersion. Thus, the results support a community-level compositional response to drought, although they do not establish statistically significant differential abundance of individual ASVs after correction for multiple comparisons. These results indicate that drought significantly altered the overall composition of the endophytic fungal community, with exploratory taxon-specific abundance patterns that may reflect ecological responses relevant to plant adaptation and stress tolerance [49].

At the family level, well-watered plants were characterized by a high relative representation of Nectriaceae, whereas drought-treated plants showed a lower observed representation of this family and higher relative representation of groups such as Aspergillaceae, together with greater proportions of unclassified and low-abundance taxa. Members of Nectriaceae are frequently reported as plant-associated endophytes, and their occurrence within plant tissues can be influenced by environmental conditions that modify plant physiology and the internal environment available for fungal colonization [50]. Similarly, Aspergillaceae, particularly members of *Aspergillus*, are frequently reported from dry or low-moisture environments. These ecological characteristics provide useful biological context for the numerical patterns observed here but do not independently establish drought-driven selection of these taxa.

Several members of *Aspergillus*, *Macrophomina*, and *Chaetomium* are capable of tolerating low water availability, elevated temperature, or oxidative stress. *Aspergillus* species, for example, include xerotolerant fungi capable of growth at low water activity, whereas *Macrophomina* is well known for its persistence under dry soil conditions and its association with drought-related plant diseases [51]. Likewise, *Chaetomium* species include robust saprotrophs capable of persisting under nutrient-limited and stressful conditions. The numerically higher representation of some of these taxa in drought-treated samples is therefore consistent with ecological characteristics reported in the literature. However, because the corresponding ASV-level differences did not remain statistically significant after BH-FDR correction, these patterns should be regarded as exploratory rather than evidence of drought-driven enrichment or selection. *Endophytic Chaetomium* sp. strain V3 has previously been isolated from ragweed (*Ambrosia artemisiifolia* L.) and shown to possess plant growth-promoting traits in tomato [52]. Other *Chaetomium* species have also been associated with beneficial plant responses under environmental stress. For example, *Chaetomium cupreum* promoted the growth of *Miscanthus sinensis* under aluminum stress [53] and was reported to regulate the expression of genes associated with metal tolerance and plant growth promotion in *Eucalyptus globulus* roots [54]. These previous observations provide a basis for further investigation of the numerical patterns observed in the present study.

The observed representation of genera such as *Aspergillus*, *Macrophomina*, and *Chaetomium* under drought may also be considered in relation to their potential opportunistic or stress-associated lifestyles. Water deficit can alter plant physiology, host metabolism, and defense responses, potentially creating conditions favorable to fungi capable of shifting among endophytic, saprotrophic, and pathogenic lifestyles [55]. For example, *Macrophomina phaseolina*, the causal agent of charcoal rot in many crops, including legumes, is well known to proliferate under high temperature and low soil moisture, with disease severity often increasing during drought [56]. Likewise, several Aspergillus species have been reported as plant-associated fungi capable of behaving as opportunistic pathogens or latent colonizers, particularly when plants experience abiotic stress [57]. In the present study, however, the observed abundance patterns alone do not demonstrate increased pathogen recruitment or disease risk, and their ecological significance requires further investigation.

At the species level, *Fusarium falciforme* was numerically more abundant in well-watered plants, while *Fusarium oxysporum* showed a similar numerical pattern. In contrast, *Aspergillus sydowii* and *Aspergillus ochraceopetaliformis* showed higher observed abundance under drought conditions. These taxon-specific patterns were not statistically significant after BH-FDR correction and therefore should not be interpreted as evidence of drought-driven enrichment, depletion, or selection. Similarly, the higher observed proportion of uncultured and rare taxa in drought-treated samples represents a descriptive pattern rather than evidence of the creation of new ecological niches. Collectively, these taxon-level observations provide hypotheses that can be tested in future studies with greater replication.

*Aspergillus sydowii* has been reported as a plant-associated species capable of tolerating low water activity and environmental stress [58]. *Aspergillus ochraceopetaliformis* has been reported as an endophytic species isolated from plant tissues, including leaves of *Anthurium brownii*, although its potential contribution to plant drought responses or growth promotion remains poorly characterized. *Penicillium citrinum* has also been reported to promote plant growth and enhance drought tolerance through mechanisms involving phytohormone production, nutrient availability, and modulation of plant stress responses [59,60]. These reports provide biological context for the nominal or numerical patterns observed in the present study. However, none of the corresponding ASV-level comparisons remained statistically significant after BH-FDR correction, and these observations should therefore be considered hypothesis-generating rather than evidence of statistically supported drought-associated differential abundance.

Metabolic functional predictions of the fungal ASVs showed saprotrophic dominance, suggesting that fungal communities primarily sustain nutrient cycling and organic matter turnover and indirectly support plant growth and soil functionality [61]. However, the coexistence of pathotrophic and symbiotrophic fungi reflects a dynamic ecological balance in which microbial interactions regulate pathogenic pressure while maintaining beneficial functions. Functional plasticity enables many endophytes to shift along the saprotroph-symbiotroph–pathotroph continuum in response to host status and environmental stress, contributing to ecosystem resilience and plant adaptation under drought or other constraints [62]. Recent studies highlight that plant–fungal interactions encompass both growth-promoting symbioses and disease-causing relationships, emphasizing the importance of microbial equilibrium in shaping plant performance [63].

The integration of culture-dependent isolation and high-throughput sequencing provides complementary insights into the structure and drought responsiveness of the cowpea endophytic mycobiome [33]. The relatively low diversity among isolates contrasts with the broader taxonomic spectrum captured by sequencing, highlighting how methodological filtering shapes inferred community composition. Culture-based approaches preferentially recover fast-growing and sporulating taxa, whereas sequencing detects both dominant and low-abundance lineages, including those that are difficult to cultivate.

In contrast, *Fusarium falciforme* showed numerically lower abundance under drought conditions. Although such a pattern could be consistent with sensitivity to drought-associated changes in host physiology or resource availability, the ASV-level difference did not remain statistically significant after BH-FDR correction and therefore cannot establish a drought response. Its recovery through both sequencing and cultivation nevertheless confirms its presence as a component of the cowpea root endophytic community. Given the potential for Fusarium species to occupy different ecological roles depending on host and environmental context, further investigation is needed to establish the ecological significance of this taxon [64].

Differences between the dominant cultured taxa and the taxa detected by sequencing further emphasize the value of combining culture-dependent and culture-independent approaches. For example, *Penicillium singorense* was frequently recovered in culture but did not show a statistically significant abundance difference in the ASV dataset. Similarly, *Aspergillus ochraceopetaliformis* and *Penicillium citrinum* were detected only in drought samples in the sequencing dataset. Although this treatment-specific detection is noteworthy, the corresponding ASV-level comparisons were not significant after BH-FDR correction and therefore cannot establish drought-driven recruitment or proliferation. These observations instead illustrate differences in detection between treatments that can be examined in future studies with larger sample sizes.

Endophytic fungal isolates showed substantial variation in plant growth-related biochemical traits, indicating functional heterogeneity within the culturable fraction of the mycobiome. ACC deaminase activity and Salkowski-reactive indolic compounds, interpreted here as presumptive IAA because the Salkowski assay is not specific to IAA, represent traits potentially relevant to plant-endophyte interactions under environmental stress [65]. Consistent with the observed functional variation, inoculation of wheat seedlings with selected endophytic fungal isolates affected shoot and root growth under PEG-induced osmotic stress compared with uninoculated controls. PEG-induced osmotic stress in Petri-dish assays differs mechanistically from soil drought, particularly in the rate and nature of water-potential changes and in the absence of soil physical and biological complexity [66,67]. Accordingly, these results provide evidence of isolate-associated effects under PEG-induced osmotic stress rather than direct evidence of enhanced drought tolerance in cowpea [68]. *Aspergillus venezuelensis* (2F) was particularly notable because it enhanced root elongation under PEG-induced osmotic stress and promoted okra pollen germination under both control and stress conditions. It was also the only isolate assigned to the highest qualitative category (++) for ACC deaminase activity among the cultured endophytic fungi (Table 4). These functional observations identify isolates of interest for further investigation but do not demonstrate enhanced drought tolerance in cowpea.

## Limitations and future directions

It should be noted that, although the fungal isolates characterized here were recovered from drought-exposed cowpea roots and exhibited plant growth-promoting traits in heterologous bioassay hosts (wheat, okra), a direct fundamental benefit of these isolates to cowpea itself under drought was not tested in the present study. Confirming this benefit by re-inoculating cowpea under controlled drought conditions is an essential next step to validate the translational potential of these isolates as cowpea-targeted bioinoculants.

While the observed growth responses in wheat seedlings demonstrate the functional bioactivity of the isolates under osmotic stress, these effects likely stem from a multifaceted interaction involving potential tissue colonization alongside the influence of fungal metabolites or cell-wall elicitors present in the initial inoculum. To further refine the mechanistic understanding of these interactions, future studies could employ isolate-specific molecular markers or advanced microscopy to quantify the relative contribution of active colonization. In this study, all isolates were recovered from rigorously surface-sterilized root tissues, a robust and widely accepted standard criterion for establishing endophytic status. Given that these septate Ascomycota (e.g., *Fusarium*, *Aspergillus*, and *Penicillium*) do not form the arbuscular structures typical of mycorrhizal fungi, their endophytic identity in this study is appropriately characterized through the integrated use of culture-dependent and molecular methods, with future research extending these findings through in situ localization to map their specific ecological niches within the root architecture. Pots were used in this study rather than individual plants as the unit of biological replication (n = 4 pots per treatment) because plants sharing a pot are not independent with respect to soil, water, and microbial exposure; while this design avoids pseudoreplication [69], it results in a comparatively modest number of independent replicates for taxon-level statistical comparisons, and the per-taxon results in Table 2 should be interpreted accordingly.

In conclusion, drought significantly altered the composition of cowpea root endophytic fungal communities, while most measures of taxonomic and phylogenetic diversity remained relatively stable. Functional prediction indicated a predominance of saprotrophic taxa, suggesting potential roles in endophytic community functioning under water limitation. Culture-based isolation and physiological assays further revealed that representative endophytes possess diverse plant growth-promoting traits, with several isolates improving wheat seedling performance under PEG-induced osmotic stress and promoting okra pollen germination under stress. Together, these findings demonstrate a community-level response of the cowpea root mycobiome to drought and identify fungal endophytes with functional traits of potential relevance to plant performance under water-limited conditions.

## Supporting information

S1 Table Taxonomy and abundance of identified ASVs in root endophytic fungal communities of cowpea under control (WP1–WP4; Replicates 1–4) and drought (DP1–DP4; Replicates 1–4) conditions. (XLSX)

S2 Table Raw absorbance values and semiquantitative classification of ACC deaminase activity and presumptive IAA production in endophytic fungal isolates. Absorbance values are presented as mean ± SD, with the negative control used as the baseline reference. ACC deaminase activity was classified as − (≤0.2177), + (>0.2177 to 0.30), or ++ (>0.30), whereas presumptive IAA production was classified as − (≤0.15), + (>0.15 to 0.20), or ++ (>0.20).
(DOCX)

S1 Fig Rarefaction curves of endophytic fungal communities under control and drought conditions. Rarefaction curves showing (A) observed amplicon sequence variant (ASV) richness, (B) Shannon diversity, (C) Gini-Simpson diversity, and (D) phylogenetic diversity (PD whole tree) as functions of sequencing depth. Curves represent individual biological replicates from the control (WP1 to WP4) and drought-treated (DP1 to DP4) groups. The progressive stabilization of the curves with increasing sequencing depth indicates that the sequencing effort was sufficient to adequately capture the observed fungal community diversity.
(PDF)

S2 Fig ACC deaminase activity of endophytic fungal isolates. Representative photograph showing the raw experimental results of ACC deaminase activity assays for the tested endophytic fungal isolates.
(TIF)
