# Supplementary material for: Drought alters cowpea root endophytic fungal communities and functional characterization reveals plant growth-promoting potential of cultured endophytes
Source: PLoS One. 2026 Nov 11;21(11):e0360553. doi: 10.1371/journal.pone.0360553 (PMC13754142; doi:10.1371/journal.pone.0360553)
Supplement: Supplementary file 1 [file pone.0360553.s003.pdf]

Rarefaction curves of endophytic fungal communities

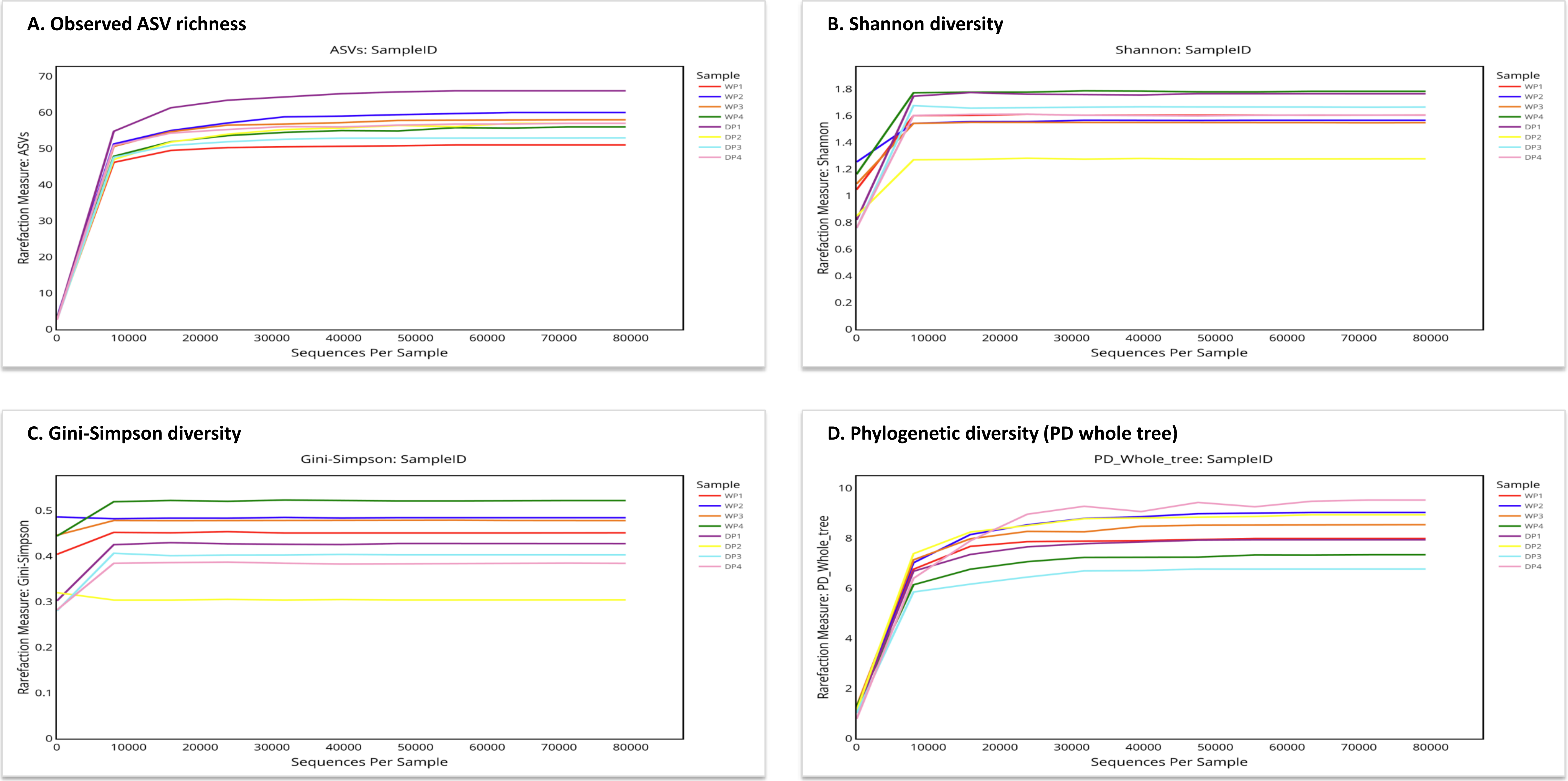

Figure S1. Rarefaction curves of endophytic fungal communities under control and drought conditions. Rarefaction curves showing (A) observed amplicon sequence variant (ASV) richness, (B) Shannon diversity, (C) Gini-Simpson diversity, and (D) phylogenetic diversity (PD whole tree) as functions of sequencing depth. Curves represent individual biological replicates from the control (WP1 to WP4) and drought-treated (DP1 to DP4) groups. The progressive stabilization of the curves with increasing sequencing depth indicates that the sequencing effort was sufficient to adequately capture the observed fungal community diversity.
