# Supplementary material for: Drought alters cowpea root endophytic fungal communities and functional characterization reveals plant growth-promoting potential of cultured endophytes
Source: PLoS One. 2026 Nov 11;21(11):e0360553. doi: 10.1371/journal.pone.0360553 (PMC13754142; doi:10.1371/journal.pone.0360553)
Supplement: Supplementary file 2 [file pone.0360553.s002.docx]

**Table S2.** Raw absorbance values and semiquantitative classification of ACC deaminase activity and presumptive IAA production in endophytic fungal isolates. Absorbance values are presented as mean ± SD, with the negative control used as the baseline reference. ACC deaminase activity was classified as − (≤0.2177), + (>0.2177 to 0.30), or ++ (>0.30), whereas presumptive IAA production was classified as − (≤0.15), + (>0.15 to 0.20), or ++ (>0.20).

| **Isolate** | **ACC deaminase (Mean ± SD)** | **Score** | **IAA (Mean ± SD)** | **Score** |
| --- | --- | --- | --- | --- |
| 1F | 0.2760 ± 0.0069 | + | 0.2307 ± 0.0467 | ++ |
| 2F | 0.3253 ± 0.0075 | ++ | 0.1323 ± 0.0320 | − |
| 3F | 0.1887 ± 0.0012 | − | 0.1417 ± 0.0747 | − |
| 4F | 0.1747 ± 0.0042 | − | 0.1140 ± 0.0178 | − |
| 6F | 0.2723 ± 0.0231 | + | 0.1773 ± 0.0158 | + |
| 7F | 0.2043 ± 0.0061 | − | 0.1307 ± 0.0309 | − |
| 8F | 0.2033 ± 0.0042 | − | 0.1343 ± 0.0245 | − |
| 9F | 0.2320 ± 0.0098 | + | 0.1357 ± 0.0502 | − |
| 10F | 0.1980 ± 0.0166 | − | 0.1440 ± 0.0250 | − |
| 11F | 0.2267 ± 0.0055 | + | 0.1417 ± 0.0428 | − |
| 12F | 0.1820 ± 0.0075 | − | 0.1410 ± 0.0214 | − |
| 13F | 0.1953 ± 0.0075 | − | 0.1347 ± 0.0389 | − |
| 14F | 0.2730 ± 0.0148 | + | 0.1750 ± 0.0157 | + |
| Control | 0.2177 ± 0.0025 | − | 0.1427 ± 0.0425 | − |
